# Simultaneous Determination of Multiclass Cyanotoxins in Aquatic Products, Vegetables and Algal Dietary Supplements Using Dispersive Solid-Phase Extraction (dSPE)-UHPLC-MS/MS

**DOI:** 10.3390/toxins18030132

**Published:** 2026-03-06

**Authors:** Baiyu Lai, Guanxiang Yuan, Qing Luo, Xiaoyun Qin, Zhaoying Lv, Haojia Ma, Huiling Chen, Honghe Liu, Guihua Liu, Jie Jiang

**Affiliations:** 1 School of Public Health, Shanxi Medical University, Taiyuan 030600, China; 2 Shenzhen Center for Disease Control and Prevention, Shenzhen 518055, China

**Keywords:** cyanotoxin, dispersive solid-phase extraction, foodstuff, microcystins, cylindrospermopsin

## Abstract

Cyanotoxins, prevalent in eutrophic aquatic ecosystems, pose significant health risks via contaminated food, yet analytical methods for detecting multiple toxin classes in foodstuffs remain limited. In the current study, a method based on dispersive solid-phase extraction (dSPE) coupled with ultra-high-performance liquid chromatography–tandem mass spectrometry (UHPLC-MS/MS) was developed to simultaneously determine four common classes of cyanotoxins (i.e., microcystins, cylindrospermopsins, nodularins, and anatoxins) in aquatic products, vegetables and algal dietary supplements. Following initial extraction with 80% aqueous methanol, sample purification was performed using anhydrous sodium sulfate (400 mg) and C_18_ sorbent (50 mg). For plant-based foods, additional graphitized carbon black (GCB, 15 mg) was also included. The method was successfully validated in eight different food matrices, demonstrating recoveries of 65–122% and relative standard deviations below 19%. The limits of detection and quantification across all matrices ranged from 0.1 to 3.4 μg/kg dry weight (dw) and 0.4 to 11.4 μg/kg dw, respectively. When applied to 96 food samples, this method detected multiple cyanotoxins in Tilapia and *Spirulina*-based dietary supplements. The proposed method provides a rapid, cost-effective, high-throughput, and sensitive analytical tool suitable for monitoring multiple cyanotoxins in various foodstuffs.

## 1. Introduction

Driven by human activities and climate change, water eutrophication has become an increasingly severe threat in global aquatic ecosystems, impacting nearly 40% of freshwater lakes [1]. Cyanotoxins, the secondary metabolites of cyanobacteria, are ubiquitous in eutrophic aquatic environments and pose significant health risks to humans via contaminated water and foodstuffs [2]. The predominant cyanotoxins found in freshwater lakes worldwide include microcystins (MCs, 63%), cylindrospermopsins (CYNs, 10%), anatoxin-a (ANA-a, 9%), and nodularin (NOD, 2%) [3]. In particular, MCs have been reported as the most prevalent cyanotoxins in 869 freshwater systems across Africa, Europe, and Asia, with levels reaching up to several milligrams per liter [3,4]. Due to their ubiquitous presence in algal blooms, environmental persistence, and multiple adverse health effects (e.g., hepatotoxicity, immunotoxicity, neurotoxicity) [5,6], several cyanotoxins are subject to regulation. As early as 2010, microcystin-LR (MC-LR) was classified as a Group 2B carcinogen by the World Health Organization (WHO) [7] In 2014, the U.S. Environmental Protection Agency (USEPA) designated ANA-a as a candidate pollutant under the Safe Drinking Water Act [8]. In 2021, NOD was included in the list of Group 3 carcinogens by the International Agency for Research on Cancer (IARC), owing to its similar hepatocarcinogenic toxicity [9] to that of MCs. CYN is also considered a potential carcinogen due to its tumor-promoting properties [10]. Some researchers have proposed a drinking water limit of 1 µg/L for CYN, based on a lowest-observed-adverse-effect level (LOAEL) of 30 µg/kg [11]. Thus, it is essential to identify the exposure risks of these cyanotoxins to human health.

Although drinking water is a primary source of human cyanotoxin exposure, other routes, especially contamination of foodstuffs, warrant consideration [2]. Several studies [12,13,14,15] have revealed that a variety of food items can accumulate cyanotoxins from contaminated water, such as aquatic animals (e.g., fish and shrimp), crops and vegetables irrigated by contaminated water, and other algae coexisting with toxin-producing species. For aquatic animals, MCs (i.e., MC-LR, MC-RR) and CYN were detected in perch muscles collected from a Canadian river during a cyanobacterial bloom, with concentrations ranging from 0.16 to 9.2 µg/kg wet weight (ww) and 45 to 75 µg/kg ww, respectively [16]. Tatter et al.’s survey [17] demonstrated that approximately half of the mussel samples along the California coastline contained multiple MCs (i.e., MC-RR, MC-dmLR, MC-YR, MC-LR, and MC-LA), with total concentrations as high as 232 µg/kg ww. Vegetables and crops can be contaminated through irrigation with cyanotoxin-rich water [13,18]. For example, lettuce and rice irrigated with Lake Taihu (China) water sampled during natural algal blooms were found to accumulate MCs (MC-YR, MC-RR, MC-LR) at levels of 3.6–424 μg/kg dry weight (dw) and 1.4–1504 μg/kg dw. However, the extent of cyanotoxin bioaccumulation capacity differs among different species and their edible parts. Notably, a systematic review by Zhang et al. [19] found that rice, dill, cabbage, radish, corn, beans, durum wheat, lettuce and carrots exhibited moderate to high bioaccumulation capacity for MCs. Some algae, such as *Spirulina* and *Chlorella* [20], commonly used for dietary supplements, can coexist with other toxic cyanobacterial species like Microcystis in freshwater environments. These toxic algae may release cyanotoxins into surrounding waters, thereby contaminating the non-toxic algae [21]. Vichi et al. [20] reported that MCs were detected in 65% of commercial algal dietary supplements collected in Italy, with MC-LR equivalent concentrations ranging from 70 to 520 µg/kg dw. Therefore, it is crucial to monitor cyanotoxins in food, especially given their inherent toxicity and potential to interact with other factors, thereby exacerbating health risks [22,23]. However, research on cyanotoxin residues in these foodstuffs remains limited, with most studies focusing on MCs rather than the broader range of cyanotoxin classes [24,25,26] that are commonly found coexisting in environmental water bodies [27,28].

Various detection technologies for cyanotoxins have been reported, including enzyme-linked immunosorbent assay [26], high-performance liquid chromatography [29] and ultra-high-performance liquid chromatography–mass spectrometry (UHPLC-MS/MS) [30]. Among these, UHPLC-MS/MS is widely employed due to its high selectivity, sensitivity, and throughput. Owing to the presence of numerous interferences (e.g., proteins, fats, vitamins and pigments) in food samples, further purification is usually required following sample extraction. Several methods, including direct dilution [31], solid-phase extraction (SPE) [32], and dispersive solid-phase extraction (dSPE) [33], have been developed for cyanotoxin detection in various food matrices. The direct dilution method is simple, rapid, and cheap, while its applicability is limited to samples with high cyanotoxin residues (e.g., samples from algal blooms) or those analyzed using highly sensitive instruments [34]. The SPE method is widely applied in the purification of MCs in food samples [35,36], but its application to multiple cyanotoxin classes involves a considerably more complex procedure, due to their diverse physicochemical properties. Aparicio-Muriana et al. [35] described a tandem column method using Strata-X and MCX to simultaneously analyze ANA, NOD, and MCs in algal dietary supplements. Díez-Quijada Jiménez et al. [36] reported a C_18_ and PGC dual-column system for the detection of MCs and CYN in mussels. Although these methods achieve acceptable recoveries, they are expensive, time-consuming, labor-intensive, and difficult to automate. Compared with the SPE method, the dSPE method is more cost-effective, faster, and operationally simpler. It has been widely applied in the detection of mycotoxins, veterinary drugs, and pesticides in routine food-monitoring programs [37,38,39]. For cyanotoxins, Qian et al. [33] utilized PSA and GCB sorbents to detect MCs in lettuce. Fang et al. [40] employed neutral alumina and GCB sorbents to analyze MCs and CYN in fish, shrimp and shellfish. However, existing dSPE methods typically focus on a single class of food matrices or cyanotoxins. A high-throughput method suitable for various food matrices remains limited for the simultaneous analysis of multiple classes of cyanotoxins.

Therefore, this study aimed to develop and validate a UHPLC-MS/MS method based on the dSPE technology for the simultaneous determination of four common cyanotoxin classes (i.e., ANA-a, CYN, NOD and MCs) in aquatic products, vegetables and algal dietary supplements. This method provides accurate and reliable technical support for the rapid quantification of multiple cyanotoxin species in diverse food items for food monitoring programs.

## 2. Results and Discussion

### 2.1. Optimization of Instrumental Conditions

Although mass spectrometric parameters (e.g., precursor ions and product ions) for all target cyanotoxins have been reported [35,41], establishing locally optimized parameters is still necessary. This is because the reported multiple-reaction-monitoring (MRM) parameters vary considerably across different studies, particularly for MCs, leading to confusion for readers. To address this problem, we separately established and optimized the MRM transitions for the target cyanotoxins on two instrument brands (AB SCIEX QTRAP^®^ 6500+ and Shimadzu 8060NX). As presented in Tables S1 and S2, the MRM transitions for ANA-a and CYN remained consistent between the two instruments, and also aligned with previously reported studies [35,42]. NOD produced similar precursor ions and product ions across both instruments, but the selection of the quantitative ion was based on its relative abundance on each respective instrument. For MC-LA and MC-LF, despite the consistent precursor ions ([M + H]^+^), their secondary fragmentation patterns differed significantly between the AB 6500+ and Shimadzu 8060NX, leading to variations in product ions. For other MC variants, precursor ions could appear as either single-charged [M + H]^+^ adducts or double-charged [M + 2H]^2+^ adducts in positive mode, but the relative abundance of these adducts varied between the two instruments (Table S3). Notably, for MC-LW, -HilR, and -LY, the [M + 2H]^2+^ adducts were significantly more sensitive on the AB 6500+ compared to their [M + H]^+^ adducts, with an inverse trend observed on the Shimadzu 8060NX. In contrast, for the remaining six MC variants (i.e., MC-RR, -YR, -LR, -WR, -HtyR, and [D-Asp3]MC-LR), [M + 2H]^2+^ precursor ions were dominant with both instruments. These results clearly demonstrated that all reported MRM transitions for cyanotoxins could only be used for reference, except for the same or similar instrument type. In addition, for most MCs, *m*/*z* 135 was routinely employed as the quantitative ion, regardless of the precursor ion pattern, due to its high sensitivity. For the LC conditions, while normal-phase chromatography columns (e.g., HILIC and amide columns) offered improved retention for the two hydrophilic cyanotoxins ANA-a and CYN, they failed to provide sufficient chromatographic separation for the MC variants and NOD. Therefore, a 15 cm C_18_ column was ultimately selected for the separation of the fourteen target cyanotoxins. Typically, an initial mobile-phase organic proportion of 5% was employed, as higher proportions led to significant solvent effects for ANA-a and CYN, causing aberrant peak shape and peak splitting.

### 2.2. Optimization of Extraction Conditions

**Figure 1:**
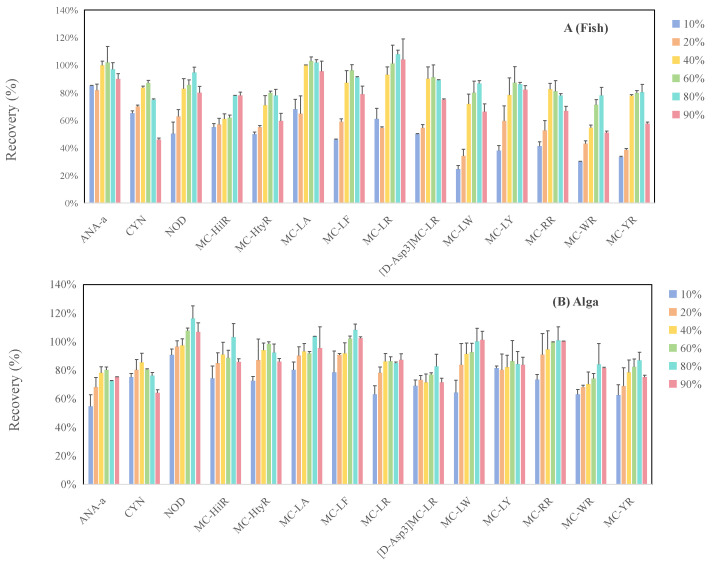
Comparison of cyanotoxin extraction efficiencies from fish (**A**) and alga (**B**) using different methanol/water solutions.

**Figure 2:**
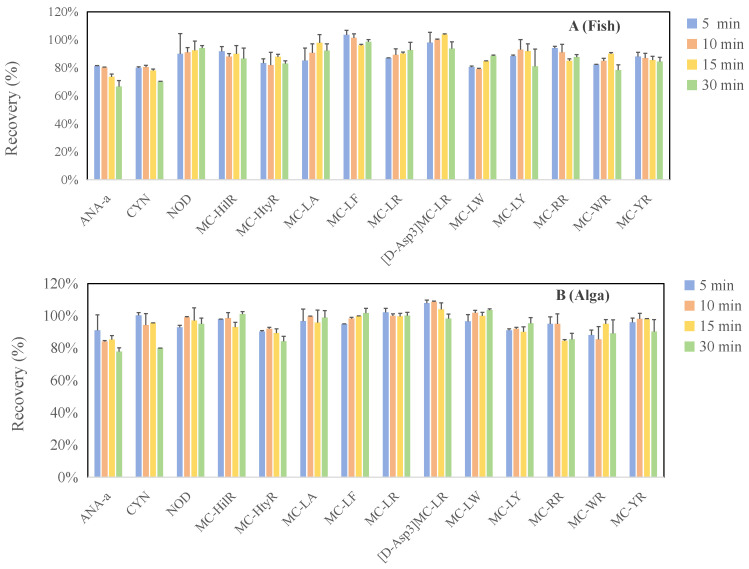
Comparison of cyanotoxin extraction efficiencies from fish (**A**) and alga (**B**) with different sonication times.

**Figure 3:**
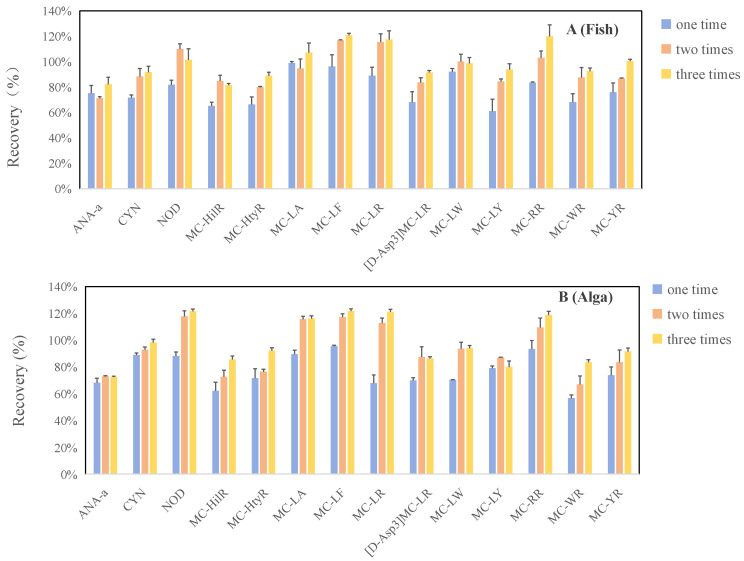
Comparison of cyanotoxin recoveries from fish (**A**) and alga (**B**) with different extraction cycles.

The optimization of sample preparation conditions for cyanotoxins in food matrices was conducted using *Spirulina*-based dietary supplements and Crucian carp as representative plant-based and animal-based food matrices, respectively. Extraction conditions, including extraction solvent, ultrasound duration and number of extraction cycles, were systematically explored. For extraction solvents, six methanol–water mixtures at varying proportions (10%, 20%, 40%, 60%, 80%, and 90%) were compared, given the widespread application of methanol-rich solutions (50–100%) for extracting cyanotoxins from diverse food matrices. As shown in Figure 1, MCs and NOD exhibited the highest extraction efficiency with an 80% methanol–water solution, which aligns with most reported studies on vegetables and algal dietary supplements [41,43,44]. The relatively lower methanol concentration compared to some aquatic product extraction methods might be attributed to the high moisture content of the fresh samples used in their studies [36,40]. For ANA-a and CYN, the extraction efficiencies in Crucian carp and *Spirulina* samples were the highest at 40–60% methanol in water, showing a declining trend beyond 60% methanol–water. This is likely due to their greater hydrophilicity compared to MCs and NOD. Thus, the 80% methanol–water solution was ultimately selected as the optimal extraction solvent. Within the 5–15 min interval, ultrasound duration exerted limited influence on sample extraction efficiency for the target cyanotoxins (Figure 2). Regarding extraction cycles, ANA-a displayed minimal sensitivity to extraction frequency, yielding good recovery with a single extraction (Figure 3). However, CYN, NOD, MC-LF, MC-LR, [D-Asp3]MC-LR, and MC-LW required at least two extractions for significantly enhanced recovery. Substantial toxin recovery was achieved with each extraction for MC-HtyR and MC-YR, thus necessitating three cycles. As for MC-LY, MC-HilR, MC-WR, and MC-LA, optimal extraction efficiency in Crucian carp and Spirulina was achieved after 2–3 extraction cycles, respectively. Therefore, considering all the aforementioned results, using three extractions is generally recommended.

### 2.3. Optimization of Purification Conditions

**Figure 4:**
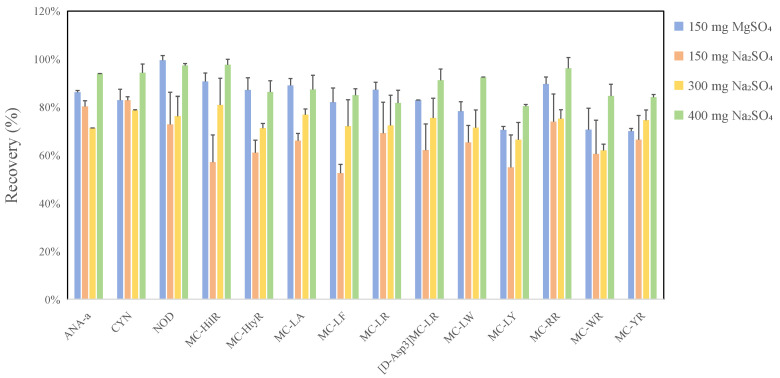
Comparison of cyanotoxin recoveries with different drying agents.

Compared to the traditional SPE method, dSPE technology achieves improved sample purification by maximizing contact area between the sorbent and target compounds through dispersing the solid sorbent into the sample matrix. Thus, the efficiency of the dSPE process primarily relies on the selection of appropriate sorbent materials and their optimal amounts. For the water absorbents, we compared the dehydration efficiencies and potential adsorption of target cyanotoxins by the two most common drying agents, anhydrous magnesium sulfate (MgSO_4_) and anhydrous sodium sulfate (Na_2_SO_4_). The examined amount of these absorbents was selected based on typical usage recommendations in commercial products. The testing samples were prepared within a 1 mL volume of 80% aqueous methanol solution. The results revealed that MgSO_4_ exhibited a significantly higher dehydration capacity than Na_2_SO_4_, with 150 mg proving as effective as 400 mg of Na_2_SO_4_ for drying extracts. Nevertheless, this amount of MgSO_4_ also inadvertently adsorbed some methanol, resulting in extraneous volume loss in addition to water removal. In terms of toxin adsorption, 150 mg of MgSO_4_ demonstrated significantly higher adsorption of cyanotoxins (e.g., ANA-a, CYN, MC-[D-Asp3] RR, MC-LY, MC-RR, MC-WR, and MC-YR) than did 400 mg of Na_2_SO_4_ (Figure 4). Therefore, 400 mg anhydrous sodium sulfate was ultimately chosen for its adequate dehydration capacity and limited impact on the adsorption of target cyanotoxins.

**Figure 5:**
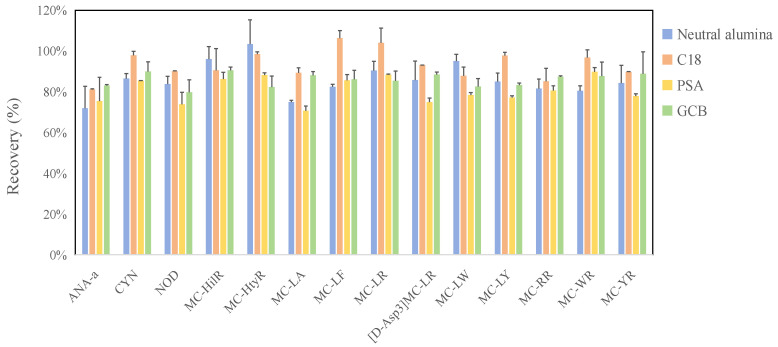
Comparison of cyanotoxin recoveries with different adsorbents.

To identify the optimal adsorbent for dSPE, four commonly used materials were evaluated, including 50 mg C_18_, 50 mg PSA, 15 mg GCB, and 50 mg neutral alumina. These adsorbents exhibit distinct properties for impurity removal: C_18_ is effective for non-polar impurities (e.g., fats), PSA targets polar impurities (e.g., carbohydrates, phenols) and carboxyl-containing compounds, GCB is suitable for pigments, and neutral alumina is employed for mild degreasing [40,45]. The ideal adsorbent should selectively adsorb major impurities from the extract while excluding the target cyanotoxins. Thus, we first investigated the adsorption of these materials on the 14 cyanotoxins. As illustrated in Figure 5, PSA exhibited significant adsorption for most cyanotoxins, and neutral alumina also showed some adsorption for certain cyanotoxins (e.g., ANA-a and MC-LA). Meanwhile, C_18_ achieved the highest recovery rates for nearly all target cyanotoxins (80–106%), followed by GCB (80–90%). Thus, C_18_ was selected as the alternative adsorbent. To evaluate its impurity removal capacity and determine the optimal sorbent amount, comparative experiments were conducted using varying amounts of C_18_ sorbent (20, 30, 50, and 100 mg) applied to *Spirulina*-based dietary supplements and Crucian carp extracts. Matrix clarity was achieved only at sorbent amounts of 50 mg and above for both sample matrices. The majority of target cyanotoxins exhibited the highest recoveries at a 50 mg sorbent dose, except for MC-LY in Crucian carp and MC-LR, [D-Asp3]MC-LR, and MC-LY in *Spirulina*. Consequently, a C_18_ sorbent dose of 50 mg was selected as the optimal amount. Additionally, obvious interferences from pigments were observed in *Spirulina*-based dietary supplement samples, manifesting as unclearness in concentrated samples. This interference may lead to blockages in instrument tubing and injection needles. To address this, an additional 15 mg of GCB was incorporated into the aforementioned dSPE materials for plant-based food matrices, without significantly compromising their high recoveries. Overall, the purification protocol using 400 mg anhydrous sodium sulfate and 50 mg C_18_ exhibited optimal performance for 14 cyanobacterial toxins in aquatic products. For algal dietary supplements, the incorporation of an additional 15 mg of GCB was necessary.

### 2.4. Resuts of Method Validation

**Figure 6:**
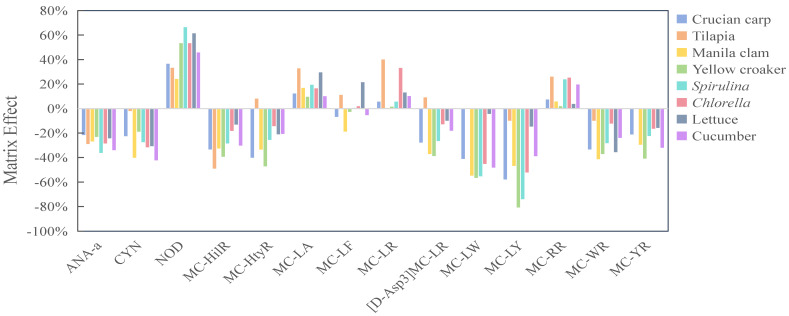
Matrix effects of cyanotoxins in different food matrices with the dSPE method.

Validation of the established method was performed on several representative food matrices, including Crucian carp, Tilapia, Manila clam, Yellow croaker, lettuce, cucumber, *Spirulina*-based dietary supplements and *Chlorella*-based dietary supplements. Matrix effects (MEs), linearity, accuracy, precision and sensitivity were assessed using the method detailed in Section 4.5. As illustrated in Figure 6, most of the target cyanotoxins (i.e., ANA-a, CYN, MC-HtyR, MC-HilR, [D-Asp3]MC-LR, MC-LY, MC-LW, MC-WR and MC-YR) displayed significant matrix suppression, with the ME values ranging from −81% to −21%. In contrast, NOD displayed pronounced matrix enhancement (MEs: 24–66%). For MC-LA, MC-LF, MC-LR and MC-RR, acceptable matrix effects were observed in most of the investigated matrices, but remained significant in some specific matrices, such as MC-LA and MC-LR in Tilapia. These results indicated that although the dSPE sorbents could reduce several interferences for cyanotoxins originating from various food matrices, the matrix effects could not be completely eliminated. This is consistent with many previous dSPE methods for pesticide and veterinary drug residues [37,46], where matrix-matched calibration curves are commonly employed. Therefore, to eliminate matrix interference and achieve precise quantification, eight matrix-matched calibration curves were established for each target cyanotoxin. All these calibration curves for the 14 cyanotoxins exhibited good linearity within their linear ranges (0.5–50 µg/L), with R^2^ values greater than 0.99. Based on these calibration curves, the recoveries of the 14 cyanotoxins were 70–115% for aquatic products, 65–122% for vegetables, and 70–120% for algal dietary supplements (Table 1). Relative standard deviations (RSDs) for all cyanotoxins at each spiking level were below 19% across all food types (Table 1). The method’s limits of detection (LODs) and quantitation (LOQs) in all eight investigated food matrices ranged from 0.1 to 3.4 µg/kg dw and 0.4 to 11.4 µg/kg dw, respectively (Table 2). The developed method’s performance parameters were comparable to those previously reported, with particularly significant improvement in sensitivity compared to many other methods (Table 3). The higher LOD observed, relative to the method reported by Leticia D.Q. et al. [47], may be attributed to their limited classes of cyanotoxins and food matrix types. Furthermore, this method is rapid, simple to operate, and suitable for high-throughput monitoring of four common cyanotoxins in food samples (Table S4). It should be noted that, consistent with most previous methods, the cyanotoxins determined in foodstuffs by the present method were in their free form. Covalently bound forms of microcystins have been reported in animal and plant matrices [48].

### 2.5. Application to Real-Food Samples

The validated method was subsequently applied to the investigation of 96 food samples, including 89 commercial and 7 field samples. As shown in Table 4, among the eight food categories, only two *Spirulina*-based dietary supplements (2/48) and four Tilapia (4/9) contain detectable certain cyanotoxins. For Tilapia, all three samples obtained from the lake displayed significant contamination with multiple cyanotoxins. CYN was the primary toxin, with levels of 9.0–14.5 µg/kg dw, followed by MC-LY (3.4 µg/kg dw), [D-Asp3]MC-LR (1.5–1.8 µg/kg dw), MC-LR (0.4–3.1 µg/kg dw), and MC-LW (0.5 µg/kg dw) (Table 4). This toxin profile in fish was highly consistent with the typical cyanotoxin characteristics of the respective lake water [54]. Although MC levels in our investigation were much lower than those found in eutrophic water bodies [55,56,57], the CYN concentrations were higher than those in Berry’s study [58]. In contrast to lake-caught specimens, only one market-collected Tilapia sample contained limited MC-LR (0.6 µg/kg dw). This is likely due to strict environmental controls during aquaculture. Furthermore, the absence of cyanotoxins in lake-caught Crucian carp may be attributed to varying toxin accumulation capacities among different fish species [59,60]. For *Spirulina*-based dietary supplements, CYN and MC-LR were detected in two separate samples at levels of 0.9 µg/kg dw and 0.5 µg/kg dw, respectively (Table 4). This detection rate and these levels were comparable to most previous investigations of *Spirulina*-based dietary supplements [61,62,63], with the exception of two studies reported in China (2008) and Canada (2017) [53,64]. It is noticed that the majority of positive findings for algal dietary supplements were associated with *Aphanizomenon*-based products [43,63]. In these products, MCs and ANA-a were detected at levels of 70–5800 µg/kg. However, *Aphanizomenon*-based products are seldom available in the Chinese market, with *Spirulina* being the predominant algal dietary supplement. In the present study, none of the 14 cyanotoxins were detectable in the investigated vegetables (i.e., lettuce and cucumber). One potential explanation is that cucumber and lettuce are generally classified as vegetables with low to moderate capacity for cyanotoxin bioaccumulation [19]. A second plausible factor is that the market-sourced vegetables may not have been irrigated with contaminated water.

## 3. Conclusions

In the present study, we developed and validated a rapid, simple, high-throughput, sensitive, and cost-effective UHPLC-MS/MS method based on dSPE technology for the simultaneous determination of four cyanotoxin categories in aquatic products, vegetables, and algal dietary supplements. The method performance was comparable or superior to prior SPE-based methods, with the extra advantages of broader food matrix applicability and cyanotoxin coverage. This method was successfully applied to 96 real-food samples, and multiple cyanotoxins (including CYN and MCs) were detected in freshwater fish and algal dietary supplement samples. Therefore, this method is proposed as a reliable tool for the monitoring of multiple cyanotoxin residues in various food items in the future.

## 4. Materials and Methods

### 4.1. Reagents and Chemicals

Standards of fourteen cyanotoxins, including MC-RR, MC-LR, MC-LY, MC-LA, MC-LW, MC-YR, MC-HtyR, MC-LF, MC-WR, MC-HilR, [D-Asp3]MC-LR, NOD, ANA-a, and CYN, were supplied by CFW Laboratories, Inc. (Newark, DE, USA). Acetonitrile and methanol (HPLC grade) were obtained from Merck KGaA (Darmstadt, Germany). Ultrapure water was produced by the Milli-Q^®^ Advantage A10 pure water system (Darmstadt, Germany). Octadecylsilyl (C18, 40–60 μm), graphitized carbon black (GCB, 100–300 mesh), and primary secondary amine (PSA, 40–60 μm) sorbents were purchased from the Biocomma company (Shenzhen, China). AR-grade sorbent of neutral alumina (100–200 mesh) was obtained from Sinopharm Chemical Reagent Co., Ltd. (Shanghai, China). LC-MS-grade formic acid, AR-grade anhydrous magnesium sulfate (MgSO_4_) and anhydrous sodium sulfate (Na_2_SO_4_) were obtained from ANPEL Laboratory Technologies Inc. (Shanghai, China).

### 4.2. Sample Collection and Preparation

Twenty-seven aquatic products (5 Crucian carp, 6 Tilapia, 8 Manila clam, 8 Yellow croaker), fourteen vegetables (9 lettuces, 5 cucumbers) and forty-eight algal dietary supplements (45 *Spirulina*, 3 *Chlorella*) were randomly collected from local markets in Shenzhen, China, and Chinese online platforms. Another seven samples (2 Crucian carp, 3 Tilapia, 2 cucumbers) were obtained from a lake in South China. For aquatic products and vegetables, edible portions were collected, chopped into small pieces and then homogenized. The resulting homogenates were lyophilized, followed by grinding into a fine powder. Algal dietary supplement samples were processed according to formulation type: tablets were pulverized, while capsules were carefully opened and their powdered content collected for subsequent analysis. All processed samples were stored in a −20 °C freezer before analysis.

### 4.3. Sample Extraction and Purification

Approximately 0.1 g of the powdered sample was weighed and mixed with 0.8 mL of 80% (*v*/*v*) methanol in water. The sample was vortexed for 1 min, then ultrasonicated for 5 min. Subsequently, the sample was centrifuged at 15,000 rpm for 10 min, and the supernatant was collected into a 1.5 mL centrifuge tube. An additional 0.4 mL of 80% (*v*/*v*) methanol in water was added to the residue, and the extraction procedure was repeated twice. All the supernatants were combined and vortexed for homogenization. Then, 1 mL of the combined supernatant was added to a purification tube containing 400 mg of anhydrous Na_2_SO_4_ and 50 mg of C_18_ sorbent. For plant-based samples, an additional 15 mg of GCB sorbent was also added to the purification tube to remove pigments. Following vortexing for 1 min and centrifugation at 12,000 rpm for 5 min, the supernatant was collected and evaporated to near dryness under a stream of nitrogen at 40 °C. The residue was re-dissolved in 0.5 mL of 20% methanol aqueous solution, ultrasonicated for 5 min, and centrifuged again at 12,000 rpm for 5 min. The final supernatant was transferred into a 1.5 mL vial for instrumental analysis.

### 4.4. UHPLC-MS/MS Analysis

Target cyanotoxins in the samples were analyzed using an AB SCIEX QTRAP^®^ 6500+ triple-quadrupole tandem mass spectrometer coupled to a Shimadzu LC-40A liquid chromatograph (SCIEX, Framingham, MA, USA). Detailed instrumental conditions have been described in our previous study [54]. Briefly, toxin separation was achieved using an ACQUITY UPLC^®^ BEH C_18_ column (150 mm × 2.1 mm, 1.7 μm) maintained at 40 °C. The mobile phases consisted of water (A) and acetonitrile (B), both containing 0.1% (*v*/*v*) formic acid. The initial mobile-phase B proportion was 5%, and the total gradient elution program spanned 10 min. A 2 μL aliquot was injected. Data acquisition was performed using an electrospray ionization (ESI) in positive-ion mode with MRM. The MRM transitions for the 14 cyanotoxins are detailed in Table S1. The mass spectrum parameters, namely the gas curtain pressure, ion source temperature, ion spray voltage, nebulizer gas and drying gas, were set at 35 psi, 300 °C, 5500 V, 50 psi and 60 psi, respectively.

### 4.5. Method Validation

To evaluate the matrix effects (MEs) on the target cyanotoxins in different foodstuffs, eight representative matrices were selected: two freshwater fish (*Crucian carp*, Tilapia), two marine products (Manila clam, Yellow croaker), two vegetables (lettuce, cucumber), and two algal dietary supplements (*Spirulina*, *Chlorella*). The matrix effects were quantified by subtracting 1 from the slope ratios of matrix-matched standard curves to solvent-based standard curves. MEs falling outside the range of ±20% were considered indicative of significant matrix suppression or enhancement and required further consideration [46]. Linearity was evaluated by constructing calibration curves with concentrations plotted against the peak area of target cyanotoxins. Standard solutions (0.5, 1.0, 5.0, 20.0, 50.0 μg/L) were prepared in 20% (*v*/*v*) methanol in water. If matrix effects could not be ignored, matrix-matched standard curves were applied. The accuracy and precision were evaluated by conducting a series of standard addition experiments. Blank matrices were spiked at concentrations of 5, 10, and 50 μg/kg (*n* = 6). The LODs and LOQs were calculated in low-level spiked samples using S/N ratios of 3:1 and 10:1, respectively.

## Figures and Tables

**Table 1:** Recoveries for the target cyanotoxins from different food matrices.

Compound	Spiked Level (μg/kg)	Crucian Carp		Tilapia		Manila Clam		Yellow Croaker		*Spirulina*		*Chlorella*		Lettuce		Cucumber	
		Recovery (%)	RSD (%)	Recovery (%)	RSD (%)	Recovery (%)	RSD (%)	Recovery (%)	RSD (%)	Recovery (%)	RSD (%)	Recovery (%)	RSD (%)	Recovery (%)	RSD (%)	Recovery (%)	RSD (%)
ANA-a	5	99%	2%	92%	1%	107%	13%	106%	19%	95%	8%	122%	1%	110%	4%	116%	1%
	10	86%	4%	86%	2%	91%	3%	92%	1%	97%	6%	89%	1%	84%	0%	94%	0%
	50	77%	3%	107%	0%	92%	4%	87%	0%	84%	0%	76%	1%	104%	3%	120%	1%
CYN	5	70%	1%	72%	4%	71%	1%	83%	4%	104%	5%	98%	13%	72%	4%	78%	1%
	10	83%	4%	96%	1%	94%	5%	82%	13%	110%	4%	107%	2%	74%	1%	77%	2%
	50	73%	7%	93%	0%	95%	0%	73%	6%	76%	2%	76%	4%	75%	0%	86%	2%
NOD	5	79%	13%	96%	2%	72%	3%	84%	7%	73%	3%	81%	1%	76%	1%	86%	5%
	10	95%	1%	93%	13%	73%	0%	102%	11%	97%	3%	98%	3%	91%	2%	89%	1%
	50	88%	2%	95%	8%	90%	9%	85%	4%	83%	2%	99%	0%	92%	2%	95%	4%
MC-HilR	5	91%	13%	79%	3%	74%	2%	93%	1%	74%	5%	79%	1%	75%	5%	73%	1%
	10	93%	1%	80%	11%	73%	1%	82%	11%	97%	4%	77%	2%	71%	1%	78%	2%
	50	93%	8%	92%	11%	76%	3%	76%	3%	74%	4%	80%	0%	78%	1%	89%	0%
MC-HtyR	5	73%	5%	80%	5%	74%	1%	82%	2%	73%	5%	82%	0%	70%	0%	85%	2%
	10	79%	9%	79%	5%	76%	2%	78%	1%	78%	0%	75%	1%	72%	3%	86%	0%
	50	76%	6%	74%	2%	73%	3%	74%	4%	74%	7%	75%	3%	79%	2%	83%	5%
MC-LA	5	80%	16%	94%	3%	87%	9%	102%	3%	77%	11%	74%	5%	72%	4%	72%	4%
	10	89%	2%	108%	3%	76%	2%	103%	8%	90%	2%	95%	4%	81%	3%	95%	1%
	50	100%	1%	84%	2%	87%	1%	82%	7%	90%	3%	88%	1%	94%	3%	106%	5%
MC-LF	5	86%	4%	111%	6%	76%	5%	80%	1%	88%	1%	85%	1%	75%	4%	75%	1%
	10	98%	5%	103%	4%	79%	1%	78%	4%	92%	0%	83%	1%	73%	0%	76%	1%
	50	94%	3%	108%	11%	75%	6%	80%	0%	95%	4%	77%	2%	73%	3%	92%	4%
MC-LR	5	83%	1%	78%	3%	83%	3%	92%	9%	75%	3%	83%	0%	72%	2%	92%	4%
	10	90%	3%	83%	13%	78%	3%	88%	3%	79%	1%	87%	0%	73%	1%	77%	3%
	50	87%	3%	89%	13%	72%	1%	84%	8%	83%	0%	75%	7%	79%	2%	85%	2%
[D-Asp3] MC-LR	5	91%	5%	77%	0%	80%	6%	89%	4%	69%	0%	72%	4%	74%	2%	70%	0%
	10	88%	8%	86%	11%	79%	1%	92%	10%	65%	17%	91%	2%	76%	0%	80%	0%
	50	86%	6%	92%	8%	76%	3%	82%	0%	75%	0%	95%	0%	91%	2%	89%	0%
MC-LW	5	81%	14%	90%	4%	75%	3%	90%	10%	82%	3%	102%	5%	71%	1%	74%	3%
	10	84%	4%	70%	7%	79%	1%	103%	4%	86%	0%	106%	4%	73%	1%	74%	1%
	50	84%	2%	75%	7%	77%	2%	84%	3%	90%	0%	86%	5%	74%	2%	78%	1%
MC-LY	5	90%	13%	97%	1%	77%	3%	75%	1%	98%	8%	73%	1%	76%	2%	114%	4%
	10	115%	1%	97%	13%	79%	2%	81%	1%	73%	0%	82%	11%	83%	2%	84%	4%
	50	76%	8%	70%	7%	84%	8%	93%	18%	92%	4%	91%	1%	79%	2%	82%	1%
MC-RR	5	78%	4%	81%	3%	77%	3%	82%	11%	77%	4%	83%	4%	71%	1%	71%	1%
	10	83%	5%	74%	11%	75%	5%	90%	2%	83%	0%	77%	1%	108%	9%	82%	1%
	50	74%	1%	98%	4%	82%	1%	74%	6%	75%	5%	82%	1%	90%	2%	90%	4%
MC-WR	5	90%	12%	78%	9%	79%	14%	86%	10%	74%	0%	87%	3%	74%	4%	70%	0%
	10	83%	12%	83%	14%	78%	2%	70%	0%	80%	2%	83%	2%	99%	3%	73%	4%
	50	81%	0%	77%	3%	75%	2%	74%	3%	74%	1%	76%	7%	78%	3%	71%	1%
MC-YR	5	74%	4%	100%	1%	72%	3%	80%	8%	72%	4%	88%	5%	70%	3%	72%	2%
	10	80%	8%	85%	10%	76%	3%	83%	2%	93%	3%	78%	1%	82%	1%	71%	1%
	50	88%	3%	84%	4%	74%	2%	72%	4%	74%	4%	87%	2%	83%	1%	86%	3%

**Table 2:** Linear ranges, detection limits (LODs) and quantification limits (LOQs) of cyanotoxins in different food matrices.

Compound	Linearity Range (μg/L)	Crucian Carp		Tilapia		Manila Clam		Yellow Croaker		*Spirulina*		*Chlorella*		Lettuce		Cucumber	
		LOD (μg/kg)	LOQ (μg/kg)	LOD (μg/kg)	LOQ (μg/kg)	LOD (μg/kg)	LOQ (μg/kg)	LOD (μg/kg)	LOQ (μg/kg)	LOD (μg/kg)	LOQ (μg/kg)	LOD (μg/kg)	LOQ (μg/kg)	LOD (μg/kg)	LOQ (μg/kg)	LOD (μg/kg)	LOQ (μg/kg)
ANA-a	0.5–50	0.4	1.3	0.3	1.0	1.0	3.3	2.5	8.3	1.3	4.5	0.9	3.0	1.0	3.4	0.2	0.7
CYN	0.5–50	0.8	2.8	1.1	3.8	0.5	1.5	0.8	2.5	0.8	2.6	1.3	4.4	0.6	1.9	1.2	4.0
NOD	0.5–50	3.4	11.4	1.7	5.7	3.0	10.0	3.3	11.1	1.7	5.7	2.8	9.4	2.1	7.1	3.0	10.0
MC-HilR	0.5–50	1.0	3.3	0.2	0.6	0.5	1.6	0.2	0.7	0.7	2.3	0.5	1.7	0.7	2.3	0.6	1.9
MC-HtyR	0.5–50	0.2	0.8	0.2	0.8	0.2	0.7	0.3	1.0	0.3	1.2	0.5	1.5	0.5	1.5	0.2	0.5
MC-LA	0.5–50	1.2	3.9	0.5	1.7	0.8	2.6	1.1	3.8	0.4	1.5	1.5	4.9	0.5	1.7	0.9	2.8
MC-LF	0.5–50	1.1	3.8	0.4	1.3	1.0	3.2	2.1	6.9	0.4	1.4	0.7	2.3	0.3	1.2	0.3	1.0
MC-LR	0.5–50	0.7	2.4	0.2	0.7	0.9	3.1	1.0	3.4	0.4	1.3	0.5	1.6	0.8	2.6	0.4	1.3
[D-Asp3]MC-LR	0.5–50	0.7	2.5	0.1	0.4	0.6	1.9	0.7	2.3	0.7	2.4	0.5	1.7	0.5	1.6	0.5	1.7
MC-LW	0.5–50	1.9	6.4	0.2	0.7	1.5	5.1	1.8	6.0	0.5	1.5	2.5	8.3	0.6	2.0	0.2	0.6
MC-LY	0.5–50	1.8	5.9	0.6	1.9	2.5	8.3	2.5	8.3	2.2	7.2	2.3	7.7	1.2	3.8	0.8	2.5
MC-RR	0.5–50	2.5	8.3	0.5	1.5	1.9	6.4	2.1	7.0	2.0	6.8	2.2	7.5	1.5	5.0	2.0	6.6
MC-WR	0.5–50	0.6	2.0	0.5	1.7	0.3	0.8	0.5	1.7	0.6	1.9	0.2	0.7	0.4	1.3	0.1	0.4
MC-YR	0.5–50	0.4	1.2	0.4	1.4	0.4	1.3	0.4	1.3	0.7	2.3	0.4	1.2	0.2	0.5	0.4	1.4

**Table 3:** Method performance comparison of cyanotoxins in aquatic products, algal health products and vegetables among different studies.

Sample	Toxin	Extraction Solvent	Purified Materials	LOD (μg/kg)	LOQ (μg/kg)	Recovery (%)	RSD (%)	Citation
Aquatic products								
Silver carp	CYN, NOD, MC-RR, MC-YR, MC-LR	ACN: H_2_O: FA ^a^ (89:10:1)	dSPE ^b^ (C18)	5–10 (ww ^c^)	15–40 (ww)	62.3–101.2	0.3–8.6	[49]
Mussel	MC-LR, MC-RR, MC-YR, CYN	MeOH ^d^ (0.5% FA)	SPE ^e^ (C18 and PGC)	0.01–0.39 (ww)	0.23–0.40 (ww)	70.37–114.03	/	[36]
Crucian carp, Yellow croaker	CYN, MC-LR, MC-RR	MeOH	dSPE (neutral alumina and GCB)	5 (ww)	10 (ww)	68.3–104	7.7–17	[40]
Clam	h-ATX, ATX, MC-RR, MC-YR, MC-LR, MC-LA, MC-LY, MC-LW, MC-LF	80% ACN	SPE (HLB)	0.3 (ww)	1.0 (ww)	75.5–98.8	1.5–5.4	[50]
Tilapia	MC-RR, MC-LR	85% MeOH	IAC ^f^	150 (ww)	500 (ww)	61.3–117.3	0.2–18.3	[25]
Crucian carp, Tilapia, Yellow croaker	ANA-a, CYN, NOD, MC-LR, MC-RR, MC-LY, MC-LW, MC-LA, MC-YR, MC-HtyR, MC-LF, MC-WR, MC-HilR, [D-Asp3]MC-LR	80% MeOH	dSPE (Na_2_SO_4_ and C18)	0.1–3.4 (dw ^g^)/ 0.025–0.85(ww)	0.4–11.4 (dw)/ 0.1–2.85(ww)	70–115	<16	This Study
Vegetables								
Water spinach, Lettuce, Cabbage, Choisum, Carrot, Turnip, Potato, Pumpkin, Cucumber, Eggplant	MC-LR, MC-RR, MC-YR	MeOH: H_2_O: TFA ^h^ (80:19:1)	SPE (C18 and HLB)	≤7.5 (dw)	≤25 (dw)	61.3–117.3	0.2–18.3	[51]
Lettuce	MC-LR, MC-RR, MC-YR, CYN	80% MeOH	SPE (C18)	0.06–0.42 (dw)	0.16–0.91 (dw)	41–93	6.92–21.68	[47]
Cornmeal	MC-RR, MC-LR, MC-YR	MeOH: H_2_O (1:1)	SPE (HLB)	1–5 (dw)	/	69–100	/	[52]
Tomato, Cucumber, Spinach	MC-LR, MC-RR, MC-YR, MC-LW, MC-LF	MeOH	dSPE (PSA and GCB)	13.0 (dw)	43.0 (dw)	71.9–96.5	/	[33]
Lettuce, Cucumber	ANA-a, CYN, NOD, MC-LR, MC-RR, MC-LY, MC-LW, MC-LA, MC-YR, MC-HtyR, MC-LF, MC-WR, MC-HilR, [D-Asp3]MC-LR	80% MeOH	dSPE (Na_2_SO_4_, C18 and GCB)	0.1–3.0 (dw)	0.4–10.0 (dw)	70–120	<9	This Study
Algal dietary supplements								
*Aphanizomenon flos-aquae*	ANA-a, CYN, MC-LR, MC-RR, STX, dc-STX, NeoSTX, dc-NeoSTX	MeOH (0.1 M FA)	dSPE (GCB)	0.1–40 (dw)	0.3–120 (dw)	71–119	<22	[53]
*Aphanizomenon flos-aquae, arthrospira platensis, spirulina, chlorella*	NOD, MC-LR, MC-RR, MC-LA, MC-LY, MC-LF, LC-LW, MC-YR, MC-WR, [Asp3]MC-LR, [Dha7]MC-LR, MC-HilR, MC-HtyR	80% MeOH	/	0.12–1.18 (dw)	/	80–110	0.3–3.5	[41]
*Spirulina*	MC-LA, MC-LF, MC-LR, MC-LY, MC-LW, MC-YR, MC-WR, MC-RR, NOD	80% MeOH	/	22.5 (dw)	50 (dw)	69–104	/	[43]
*Spirulina*	MC-LR, MC-RR, NOD, ANA-a	H_2_O (5% FA), 80% MeOH	SPE (Strata-X and MCX)	15–45 (dw)	50–150 (dw)	64.2–102.9	<20	[35]
*Spirulina, Chlorella*	ANA-a, CYN, NOD, MC-LR, MC-RR, MC-LY, MC-LW, MC-LA, MC-YR, MC-HtyR, MC-LF, MC-WR, MC-HilR, [D-Asp3]MC-LR	80% MeOH	dSPE (Na_2_SO_4_, C18 and GCB)	0.2–2.8 (dw)	0.7–9.4 (dw)	65–122	<19	This Study

^a^ ACN: Acetonitrile, H_2_O: water, FA: formic acid; ^b^ dSEP: dispersive solid-phase extraction; ^c^ ww: wet weight; ^d^ MeOH: methanol; ^e^ SEP: solid-phase extraction; ^f^ IAC: immunoaffinity columns; ^g^ dw: dry weight; ^h^ TFA: trifluoroacetic acid.

**Table 4:** Detection of cyanotoxins in investigated aquatic products, vegetables and algal dietary supplements ^a^.

Category	Detection Rate (%)	CYN		[D-Asp3] MC-LR		MC-LR		MC-LW		MC-LY	
		DN ^b^	Levels ^c^	DN	Levels	DN	Levels	DN	Levels	DN	Levels
Crucian carp	0% (0/7)	0	/	0	/	0	/	0	/	0	/
Tilapia	44% (4/9)	3	9.0–14.5	2	1.5–1.8	4	0.4–3.1	2	0.5	2	3.4
Manila clam	0% (0/8)	0	/	0	/	0	/	0	/	0	/
Yellow croaker	0% (0/8)	0	/	0	/	0	/	0	/	0	/
*Spirulina*	4% (2/45)	1	0.9	0	/	1	0.5	0	/	0	/
*Chlorella*	0% (0/3)	0	/	0	/	0	/	0	/	0	/
Lettuce	0% (0/9)	0	/	0	/	0	/	0	/	0	/
Cucumber	0% (0/7)	0	/	0	/	0	/	0	/	0	/

^a^ NOD, MC-HilR, MC-HtyR, MC-LA, MC-LF, MC-RR, MC-WR, MC-YR, and ANA-a were not detected in all samples. ^b^ DN: Detection number of samples. ^c^ Levels refer to the toxin levels in positive samples (μg/kg).

## Data Availability

The original contributions presented in this study are included in the article/Supplementary Material. Further inquiries can be directed to the corresponding authors.

## Supplementary Materials

The following supporting information can be downloaded at https://www.mdpi.com/article/10.3390/toxins18030132/s1. Table S1: MRM parameters of 14 cyanotoxins on AB SCIEX QTRAP^®^ 6500+; Table S2: MRM parameters of 14 cyanotoxins on SHIMADZU 8060NX; Table S3: Relative abundance of the [M + 2H]^2+^ and [M + H]+ adducts for MCs on AB SCIEX QTRAP^®^ 6500+ and Shimadzu 8060NX; Table S4: Comparison of solid-phase extraction (SPE) and dispersive solid-phase extraction (dSPE) techniques used for purification of multiple cyanotoxins in food samples.
