# Supplementary material for: Simultaneous Determination of Multiclass Cyanotoxins in Aquatic Products, Vegetables and Algal Dietary Supplements Using Dispersive Solid-Phase Extraction (dSPE)-UHPLC-MS/MS
Source: Toxins (Basel). 2026 Mar 6;18(3):132. doi: 10.3390/toxins18030132 (PMC13030168; doi:10.3390/toxins18030132)
Supplement: Supplementary file 1 [file toxins-18-00132-s001.zip › toxins-4169548-supplementary.pdf]

Supplementary Materials: Simultaneous Determination of Multiclass Cyanotoxins in Aquatic Products, Vegetables and Algal Dietary Supplements Using Dispersive Solid-Phase Extraction (dSPE)-UHPLC-MS/MS

Baiyu Lai [1,2], Guanxiang Yuan [2,*], Qing Luo [2], Xiaoyun Qin [2], Zhaoying Lv [2], Haojia Ma [2], Huiling Chen [2], Honghe Liu [2], Guihua Liu [2] and Jie Jiang [1,2,*]

Table S1. MRM parameters of 14 cyanotoxins on AB SCIEX QTRAP® 6500+

Toxin	Precursor Ion (m/z)	Product Ion (m/z)	Declustering Potential (V)	Collision Energy (V)	Retention Time (min)
ANA-a	166.0	130.9*/149.0	50	21/17	3.23
CYN	416.3	194.2*/336.0	70	48/30	2.23
NOD	825.5	103.3*/135.0	220	155/90	5.78
[D-Asp3]MC-LR	491.3	135.2*/847.6	45	14/14	5.94
MC-HilR	505.3	135.1*/875.6	50	15/14	6.03
MC-HtyR	530.6	135.1*/514.6	20	14/14	5.90
MC-LA	910.7	776.4*/375.0	35	30/37	6.85
MC-LF	986.5	478.0*/375.1	20	36/45	7.42
MC-LR	498.4	135.1*/482.5	20	14/14	5.95
MC-LW	513.3	135.1*/891.4[a]	20	13/12	7.28
MC-LY	502.0	135.1*/486.0	25	13/11	6.89
MC-RR	520.2	135.1*/620.4	20	33/37	5.54
MC-WR	534.7	134.9*/934.4	20	16/16	6.05
MC-YR	523.6	135.0*/507.5	30	15/15	5.88

*: Quantative ion.
a The quantification of MC-LW in algal dietary supplements was performed using m/z 891.4, as m/z 135.9 exhibited significant interference.

MS parameters on SHIMADZU 8060NX

Table S2 showed the MRM parameters for the targeted cyanotoxins on SHIMADZU 8060NX. Other MS parameters were as follows: nebulizing gas flow: 3 L/min; heating gas flow: 10 L/min; interface temperature: 250 °C; DL temperature: 150 °C; heat block temperature: 400 ℃; drying gas flow: 5 L/min.

Table S2. MRM parameters of 14 cyanotoxins on SHIMADZU 8060NX

Toxin	Precursor Ion (m/z)	Product Ion (m/z)	Q1Pre bias （V）	Q3Pre bias （V)	Collision Energy (V)
ANA-a	166.0	131.1*	12	11	15
	166.0	149.1	18	24	17
CYN	415.9	194.1*	20	14	35
	415.9	176.1	29	19	35
	415.9	336.1	30	18	23
NOD	825.2	135.2*	24	10	62
	825.2	226.8	24	29	70
[D-Asp3] MC-LR	491.2	135.1*	24	27	13
	491.2	847.2	19	26	15
MC-HilR	1009.2	135.1*	38	26	64
	1009.2	213.0	36	26	65
MC-HtyR	530.3	135.2*	26	10	15
	530.3	514.3	26	26	12
MC-LA	910.2	776.2*	34	24	21
	910.2	135.2	26	15	59
MC-LF	986.4	135.2*	38	24	60
	986.4	375.2	38	26	40
MC-LR	498.2	135.1*	20	26	14
	498.2	861.2	12	32	15
MC-LW	1025.2	135.0*	38	25	54
	1025.2	213.0	38	21	58
MC-LY	1002.2	135.1*	30	10	60
	1002.2	868.1	38	26	22
MC-RR	519.8	135.2*	26	23	36
	519.8	105.0	26	19	49
MC-WR	534.5	135.1*	26	10	26
	534.5	104.5	20	11	30
MC-YR	523.8	135.1*	26	25	26
	523.8	103.0	26	12	52

*: Quantative ion.

Table S3. Relative abundance of the $[M + 2H]^{2+}$ and $[M + H]^{+}$ adducts for MCs on AB SCIEX QTRAP® 6500+ and Shimadzu 8060NX

Toxin	AB 6500+		Shimadzu 8060NX	
	Ratio	Optimal precursor ion	Ratio	Optimal precursor ion
[D-Asp3]MC-LR	Only $[M + 2H]^{2+}$	$[M + 2H]^{2+}$	10	$[M + 2H]^{2+}$
MC-RR	Only $[M + 2H]^{2+}$	$[M + 2H]^{2+}$	1000	$[M + 2H]^{2+}$
MC-WR	1000000	$[M + 2H]^{2+}$	3	$[M + 2H]^{2+}$
MC-YR	100	$[M + 2H]^{2+}$	3	$[M + 2H]^{2+}$
MC-HtyR	100	$[M + 2H]^{2+}$	2	$[M + 2H]^{2+}$
MC-LR	100	$[M + 2H]^{2+}$	2	$[M + 2H]^{2+}$
MC-HilR	25	$[M + 2H]^{2+}$	0.8	$[M + H]^{+}$
MC-LY	10	$[M + 2H]^{2+}$	Only $[M + H]^{+}$	$[M + H]^{+}$
MC-LW	10	$[M + 2H]^{2+}$	0.001	$[M + H]^{+}$
MC-LA	Only $[M + H]^{+}$	$[M + H]^{+}$	Only $[M + H]^{+}$	$[M + H]^{+}$
MC-LF	Only $[M + H]^{+}$	$[M + H]^{+}$	Only $[M + H]^{+}$	$[M + H]^{+}$

Table S4. Comparison of solid-phase extraction (SPE) and dispersive solid-phase extraction (dSPE) techniques used for purification of multiple cyanotoxins in food samples

Feature	SPE	dSPE
Purification Efficiency	Satisfactory	Satisfactory
Necessary Equipment	Solid-phase extraction apparatus and pump	Centrifuge
Equipment Accessibility	Accessible; Easy to obtain	Readily Available Equipment in most laboratories
Material Accessibility	Accessible; Easy to obtain	Accessible; Easy to obtain
Estimated Cost a	\$5 - \$40 per sample	< \$1 per sample
Operation and Time-Consumed	≥ 30 min (including column activation, sample loading, washing, drying, and elution)	Approximately 6 min (including vortexing and centrifugation)

a. The costs of SPE and dSPE consumables and reagents are influenced by various factors, including cartridge/sorbent type, brand, region, and time. Here, we provide a rough cost estimate based on official pricing from Chinese vendor websites.
